# Supplementary material for: Comparison of the Biomechanical Properties between Healthy and Whole Human and Porcine Stomachs
Source: Bioengineering (Basel). 2024 Feb 28;11(3):233. doi: 10.3390/bioengineering11030233 (PMC10968203; doi:10.3390/bioengineering11030233)
Supplement: Supplementary file 1 [file bioengineering-11-00233-s001.zip › bioengineering-2879902-supplementary.pdf]

Table S1

Thickness data of porcine and human stomachs. All measures are provided as mean ± SD.

		Thickness (mm)		
		Intact	Mucosa	Muscularis
Pig	Fundus	4.14±0.98	1.43±0.34	3.16±1.00
	Corpus	5.26±0.75	2.78±0.53	2.59±0.60
	Antrum	5.17±0.84	2.00±0.33	3.70±0.75
Human	Fundus	1.93±0.61	0.98±0.22	1.14±0.38
	Corpus	2.36±0.55	1.23±0.28	1.49±0.29
	Antrum	3.16±0.71	1.20±0.35	2.06±0.51

Table S2

Statistical results of thickness comparisons between different regions of porcine and human stomachs. P<0.05 were considered statistically significant for all analyses.

		Fundus vs Corpus	Fundus vs Antrum	Corpus vs Antrum
Pig	Intact	0.000	0.000	0.220
	Mucosa	0.000	0.000	0.000
	Muscularis	0.004	0.000	0.000
Human	Intact	0.036	0.000	0.001
	Mucosa	0.001	0.078	0.130
	Muscularis	0.000	0.000	0.000

Table S3

Statistical results of thickness comparisons between different layers of porcine and human stomachs. P<0.05 were considered statistically significant for all analyses.

		Intact vs Mucosa	Intact vs Muscularis	Mucosa vs Muscularis
Pig	Fundus	0.000	0.000	0.000
	Corpus	0.000	0.000	0.000
	Antrum	0.000	0.000	0.000
Human	Fundus	0.000	0.000	0.000
	Corpus	0.000	0.000	0.004
	Antrum	0.000	0.000	0.000

Table S4

Statistical results of thickness comparisons between porcine and human stomachs. P<0.05 were considered statistically significant for all analyses.

	Intact	Mucosa	Muscularis
Fundus	0.000	0.000	0.000
Corpus	0.000	0.000	0.000
Antrum	0.000	0.000	0.000

Table S5

Elastic modulus for porcine and human stomachs. All measures are provided as mean ± SD.

			Modulus (Kpa)		
			Intact	Mucosa	Muscularis
Pig	Longitudinal	Fundus	194.684±85.430	1000.970±413.529	149.872±89.513
		Corpus	3694.590±809.566	7041.760±3002.070	6122.722±1290.145
		Antrum	1649.467±648.268	1867.147±1005.017	5513.323±1435.131
	Circumferential	Fundus	80.693±36.193	538.770±243.056	122.988±48.639
		Corpus	613.859±374.233	5442.763±1774.694	229.401±55.001
		Antrum	458.620±204.209	440.388±141.599	272.805±169.507
Human	Longitudinal	Fundus	1359.627±562.283	2295.195±1420.580	954.846±789.187
		Corpus	1131.280±532.742	1916.618±585.237	1506.245±543.273
		Antrum	1759.598±662.918	2106.937±680.677	2729.321±721.034
	Circumferential	Fundus	1519.652±1080.420	1750.177±870.972	1186.412±668.091
		Corpus	1603.627±844.211	1535.957±819.184	954.088±524.878
		Antrum	1736.509±590.717	1620.808±572.526	1447.154±643.502

Table S6

Statistical results of elastic modulus comparisons between different regions of porcine and human stomachs. *P*<0.05 were considered statistically significant for all analyses.

			Fundus vs Corpus	Fundus vs Antrum	Corpus vs Antrum
Pig	Longitudinal	Intact	0.000	0.000	0.000
		Mucosa	0.000	0.037	0.001
		Muscularis	0.000	0.000	1.000
	Circumferential	Intact	0.000	0.000	1.000
		Mucosa	0.000	1.000	0.000
		Muscularis	0.000	0.000	1.000
Human	Longitudinal	Intact	0.445	0.187	0.043
		Mucosa	0.443	0.701	0.698
		Muscularis	0.488	0.000	0.053
	Circumferential	Intact	0.489	0.489	0.436
		Mucosa	0.858	0.716	0.853
		Muscularis	0.258	0.489	0.931

Table S7

Statistical results of elastic modulus comparisons between different layers of porcine and human stomachs. $P<0.05$ were considered statistically significant for all analyses.

			Intact vs Mucosa	Intact vs Muscularis	Mucosa vs Muscularis
Pig	Longitudinal	Fundus	0.000	0.784	0.000
		Corpus	0.000	0.000	1.000
		Antrum	1.000	0.000	0.000
	Circumferential	Fundus	0.000	0.154	0.000
		Corpus	0.000	0.059	0.000
		Antrum	1.000	0.007	0.006
Human	Longitudinal	Fundus	0.072	0.423	0.013
		Corpus	0.009	0.189	0.152
		Antrum	0.323	0.010	0.083
	Circumferential	Fundus	0.387	0.666	0.173
		Corpus	1.000	0.130	0.016
		Antrum	0.489	0.745	0.863

Table S8

Statistical results of elastic modulus comparisons between different directions of porcine and human stomachs. $P<0.05$ were considered statistically significant for all analyses.

		Intact	Mucosa	Muscularis
Pig	Fundus	0.000	0.000	0.606
	Corpus	0.019	0.018	0.000
	Antrum	0.000	0.000	0.000
Human	Fundus	0.931	0.262	0.753
	Corpus	0.320	0.853	0.063
	Antrum	0.802	0.161	0.972

Table S9

Statistical results of elastic modulus comparisons between porcine and human stomachs. $P<0.05$ were considered statistically significant for all analyses.

		Intact	Mucosa	Muscularis
Longitudinal	Fundus	0.000	0.001	0.000
	Corpus	0.436	0.001	0.051
	Antrum	0.464	0.253	0.008
Circumferential	Fundus	0.000	0.000	0.000
	Corpus	0.014	0.012	0.000
	Antrum	0.000	0.000	0.000
